# RANTES and developmental defects of enamel in children: A Brazilian prenatal cohort (BRISA)

**DOI:** 10.1371/journal.pone.0284606

**Published:** 2023-07-27

**Authors:** Elisa Miranda Costa, Judith Rafaelle Oliveira Pinho, Maria da Conceição Pereira Saraiva, Cecília Cláudia Costa Ribeiro, Rosângela Fernandes Lucena Batista, Cláudia Maria Coêlho Alves, Vanda Maria Ferreira Simões, Heloisa Bettiol, Marco Antônio Barbieri, Ricardo de Carvalho Cavalli, Erika Bárbara Abreu Fonseca Thomaz

**Affiliations:** 1 Department of Public Health, Federal University of Maranhão, São Luís, Maranhão, Brazil; 2 Department of Dentistry, University of São Paulo, Ribeirão Preto, São Paulo, Brazil; 3 Department of Dentistry, Federal University of Maranhão, São Luís, Maranhão, Brazil; 4 Department of Puericulture and Pediatrics, Graduate Program in Child and Adolescent Health, São Paulo University, Ribeirão Preto, São Paulo, Brazil; 5 Department of Gynecology and Obstetrics, Faculty of Medicine of Ribeirão Preto, University of São Paulo, Ribeirão, São Paulo, Brazil; Ohio State University, UNITED STATES

## Abstract

**Objective:**

Little is known about the effect of maternal immunological factors on the etiology of developmental defects of enamel (DDE). RANTES (Regulated on Activation Normal T Cell Expressed and Secreted) is a chemokine produced by fibroblasts, lymphoid and epithelial mucosa cells in response to various external stimuli. Despite its importance for embryogenesis, RANTES expression has been demonstrated in multiple diseases characterized by inflammation, tumor and immune response, and wound healing. We hypothesized that altered levels of RANTES during pregnancy are associated with the immune and inflammatory response in women, which could lead to the occurrence of DDE *in utero* (DDE-iu), directly or mediated by preterm birth. Therefore, this study aimed to evaluate the direct and indirect effects of serum levels of RANTES in pregnant women in the occurrence of DDE-iu in children.

**Methods:**

This is a longitudinal case-control study. The mothers and their children (327) were evaluated in three moments: prenatal care, post childbirth, and when the child was between 12.3 and 36 months of age. The analysis was performed with structural equation modeling, estimating the standardized coefficient (SC), adopting α = 5%.

**Results:**

There was a direct and negative effect of RANTES on the outcome (SC = -0.137; p = 0.022). This association was not mediated by preterm birth (SC = 0.007; P = 0.551). When considering the specific types of DDE-iu, RANTES had a direct effect on hypoplasia (SC = -0.190; p = 0.007), but not on opacity (SC = 0.343; p = 0.074).

**Conclusion:**

Lower serum levels of RANTES may contribute to a higher number of teeth with DDE-iu, specifically hypoplasia. However, more evidence supported by clinical, laboratory and epidemiological studies is still needed.

## Introduction

Amelogenesis begins in utero and continues until after the first year of life [1], and the enamel keeps records of metabolic disorders during odontogenesis [2]. Therefore, events during these periods may cause developmental defects of enamel (DDE) in primary dentition [3, 4]. DDE are disturbances arising in the hard tissue matrices and their mineralization during odontogenesis, and because of the non-remodeled nature of teeth, enamel defects can provide a window into the “metabolic memory” of the developmental process through the relevant stage of the life course [2].

Disturbances during the early stages of secretion, mineralization, and maturation of dental enamel can lead to alterations in the development of this hard tissue [1]. These can be classified into two major types: hypomineralization and hypoplasia [5]. If a disturbance occurs during the secretion phase; the enamel defect is called hypoplasia, but if it occurs during the mineralization or maturation phase, it is called hypomineralization. In hypomineralization, enamel has normal thickness but is not fully mineralized; the enamel may appear opaque, creamy white, or have yellow/brown discolorations, characterized as diffuse and/or demarcated opacities [5], while hypoplasia is a quantitative defect associated with a reduction in enamel thickness [6].

The DDE etiology has not been fully explained, but it has been associated with adverse birth outcomes, such as low birth weight [7], preterm birth [8, 9], and intrauterine growth restriction [10]. However, there is no consensus about this association in the literature [11]. Socioeconomic factors [3, 12], tobacco use [13], hypertension [13], gestational diabetes [14], prenatal anti-epileptic drugs [15], gastrointestinal hookworm infection [16], and alcohol consumption [17] have been potentially considered factors associated for DDE in the primary dentition.

Despite a growing number of studies investigating the role of the maternal immune response in the occurrence of DDE [18, 19], the mechanism still remains unclear. In this sense, the study of some cytokines seems promising, such as RANTES (regulated on activation, normal T cell expressed and secreted), a cytokine belonging to a regulatory group of chemokines. Although RANTES has a consistent action in pregnancy and embryogenesis [20], it has not been studied in odontogenesis.

In the presence of increased physiological concentrations of progesterone, such as in pregnant women, RANTES synthesis by CD4+ and CD8+ T cells is also increased and can act in an autocrine fashion [20]. One of the main effects of RANTES is the induction and recruitment of regulatory T cells, playing an essential role in the progression of cancer [21] and inflammation [21]. The interaction between the levels of this chemokine and bone remodeling has also been studied [22]. However, this chemokine is quite versatile; high levels of RANTES were associated with hypertension in pregnancy [23]. Some studies showed that the RANTES promoter genotype was associated with diabetic nephropathy in type 2 diabetic subjects [24], late-onset asthma [25], atopic dermatitis [26], and AIDS progression [27].

Lower serum levels of RANTES were considered a factor associated with preterm birth and sepsis [28], and lower levels of RANTES have been found in the umbilical cord blood of late pre-term neonates and babies born via C-section compared to vaginally delivered controls [29]. RANTES affects the fetus’s immune response, development, and maturation [20]. Therefore, it is possible that during head and neck development, RANTES is involved in the formation of the oral and maxillofacial regions, acting in cellular proliferation, differentiation, migration, and apoptosis, and that alterations in RANTES during pregnancy could influence amelogenesis.

Hypomineralization in primary teeth is often associated with molar incisor hypomineralization in the permanent dentition, which can pose major aesthetics and restorative challenges, and studying the possible prenatal etiological factors for DDE is highly relevant to the control and reduction of dental caries [30, 31]. However, previous studies of the association between prenatal factors and DDE used a traditional approach of multiple regression. Therefore, in this study, the relationship between prenatal factors and DDE is explored through structural equation modeling (SEM), as this analysis allows the estimation of direct and indirect effects of multiple risk factors associated with the outcome, in addition to being a robust analysis for the evaluation of a complex multi-causality phenomenon, such as DDE. Another advantage, SEM may use maximum likelihood estimation for handling missing data [32].

We hypothesize that changes in the serum levels of RANTES during pregnancy may alter the immune and inflammatory response, increasing the chances of preterm birth and defects in enamel remodeling, potentially increasing the occurrence of DDE *in utero* (DDE-iu), specifically hypoplasia and opacity, in the primary dentition. Therefore, this study aims to analyze the direct and indirect effects of RANTES on DDE-iu, considering the common risk factors in the model.

## Materials and methods

### Study design

This is an observational, longitudinal study, with data from a case-control nested in a prospective cohort of pregnant women in the city of São Luis-MA—cohort BRISA [33, 34]. The reference population consisted of pregnant women who received prenatal care in public and private health services and were referred to the University Hospital of the Federal University of Maranhão. The study followed the STROBE guidelines.

### Ethics and study location

The study was approved by the Human Research Ethics Board of the University Hospital of Federal University of Maranhão (Nº 223/2009) and was conducted under the Helsinki Declaration. All participants signed the informed consent form.

The city of São Luis, capital of the state of Maranhão (MA), is an island on the north coast of the state, Northeast region of Brazil, one of the poorest regions of the country, where only 50% of households are connected to the sewerage network and 75% receive piped water. The Human Development Index of the city is 0.768, occupying the 249^th^ position in Brazil. Its population in 2010 was 1,014,837 inhabitants and had a per capita income of R$805.36, equivalent to $351.69 USD. Its population in 2010 was 1,014,837 inhabitants and it had a per capita income of R$ 805.36, equivalent to U$ 351.69 to $359.69 USD.

### Participants and sampling

The recruitment took place between 02/2010 and 11/2011, involving 1,447 pregnant women interviewed form the 22^nd^ to 25^th^ weeks of gestation (baseline or T0). Of these, 66 pregnant women were not interviewed at the first follow-up visits or did not answer the questionnaires. A total of 1,381 (93.94%) were followed up at the time of the baby’s birth (T1). For this study, 327 dyads (pregnant women and children) were included. In the original case-control study nested to a cohort, cases were all pregnant women whose babies were born preterm (n = 109), and a control sample, 2:1 (n = 218), was selected by simple random draw without replacement in a cohort study. This sample was evaluated when children were aged between 12.3 and 36 months of age (T2).

We estimated that the sample of 327 children would have the power of 85.29% to identify significant differences in the DDE-iu ratio between exposed and unexposed to low serum levels of RANTES chemokine, considering a type I error of 5%, 1:1 ratio between exposed and unexposed, a prevalence of 4.41% of DDE-iu among unexposed and 14.49% among exposed.

### Collection of data and variables

The explanatory variables that represented the maternal and child characteristics were collected at baseline: 1) Mother’s occupation (unskilled labor, semi-skilled labor, skilled labor, office roles, higher-level worker and administrators/managers/directors/owners); 2) Years of school education of the pregnant woman (up to 8 years, and > 8 years); 3) Family income in multiples of the monthly Brazilian minimum wage (less than 1, 1 to less than 3, 3 to less than 5 and 5 or more—quartiles); 4) Brazilian economic class (A-B being the wealthiest, C, D-E being the poorest). The instrument used to measure economic class was designed by the Brazilian Association of Research Companies (ABEP, in Portuguese) [35]. To assess the socioeconomic status, a latent variable regarding the mother was devised (economic class; education, measured as years of school education of the pregnant woman; mother’s occupation; and family income).

During the baseline (T0), blood samples (20ml) from pregnant women were collected through venipuncture by a nursing technician. Blood was centrifuged for serum separation and serum was placed in Eppendorf tubes, labeled, and stored in a freezer at -80°C and was submitted to the cytometric bead array (CBA) to determine the presence and quantity of the presence and quantity of chemokine (RANTES). All used reagents were from Merck’s Milliplex Kit (USA). The cytometry was calibrated according to the manufacturer’s recommendations. The values are reported as pg/mL. Serum levels of chemokine were categorized according to the median of the sample 7,489.00 pg/ml.

In T1, we assessed gestational age (GA) through an algorithm based on two criteria: from the ultrasound performed in the first trimester of gestation and the date of the last menstrual period (LMP). GA was categorized as: preterm (<37 gestational weeks), or not.

During T2, each child was examined in a portable dental chair, under artificial light, after tooth drying by air jets, using a WHO-621 periodontal probe and a mouth mirror (©2013 Hu-Friedy), previously sterilized and individually packaged. Five trained examiners conducted the procedures to diagnose DDE (Kappa inter-examiner > 0.8). Diagnosis of dental enamel defects was performed according to a modified version of the DDE Index proposed by the World Dental Federation [6]. DDE was characterized by the number of teeth with enamel hypoplasia, diffuse opacities, and/or demarcated opacities, according to the criteria 0 to 8 (0- normal; 1- demarcated opacity; 2- diffuse opacity; 3- hypoplasia; 4- Other defects; 5- demarcated and diffuse opacity; 6- demarcated opacity and hypoplasia; 7- diffuse opacity and hypoplasia; and 8- all three defects) [6]. It is considered of uterine origin all defect present in the occlusal/incisal third [10, 36]. In this study, three outcomes were used: number of teeth with any DDE-iu, number of teeth with hypoplasia-iu, and number of teeth with opacity-iu.

### Theoretical model

**Fig 1:**
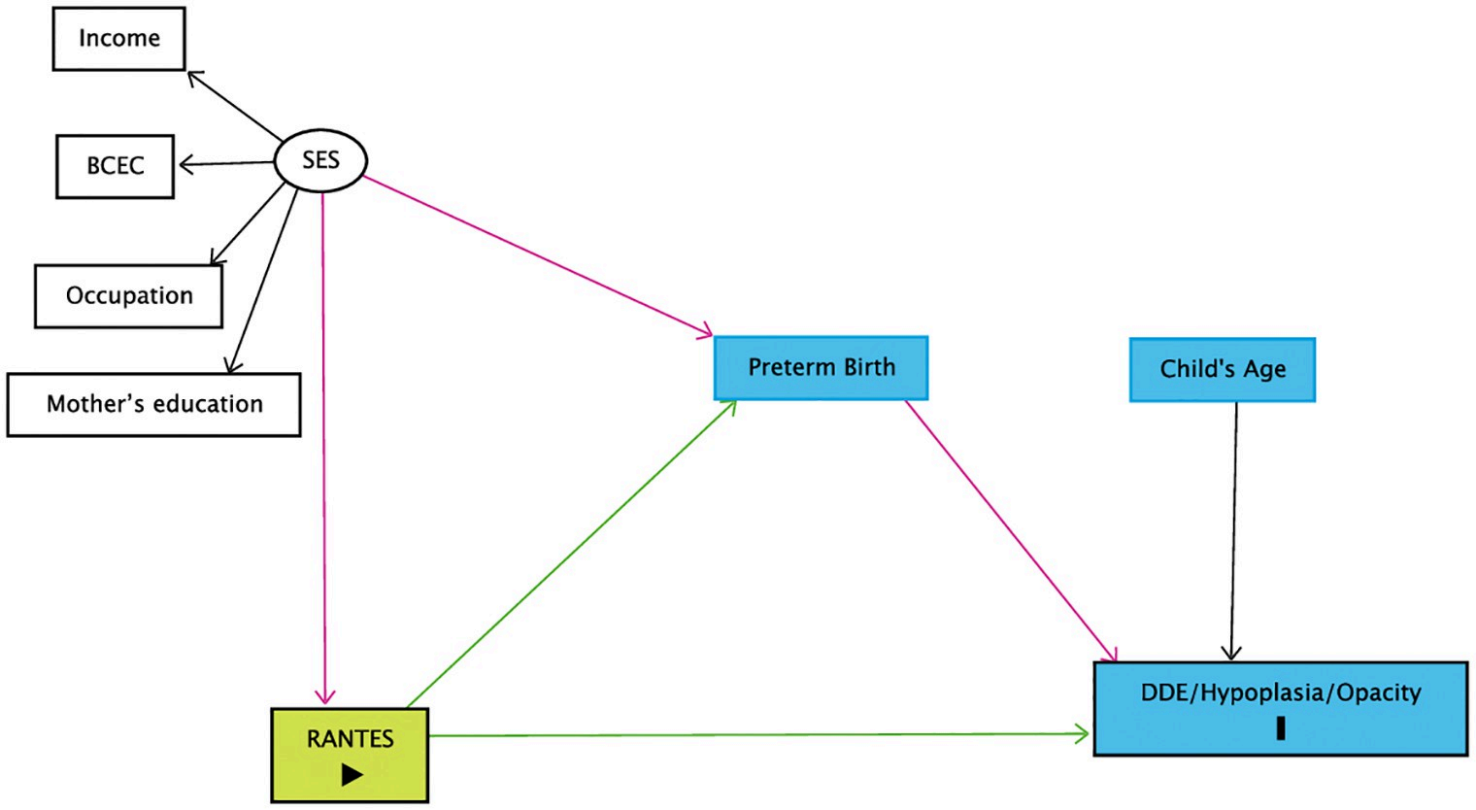
Theoretical model considering RANTES as a predictor of DDE in children from 12.3 to 36 months of age. BCEC—Brazilian Classification Economic Class. (SES) Socioeconomic Status; RANTES—Regulated on Activation Normal T Cell Expressed and Secreted; DDE—Developmental Defects of Enamel.

The theoretical model proposed suggests that unfavorable socioeconomic status would lead to a change in the maternal immune response, as assessed by RANTES during pregnancy, increasing the risk of preterm birth [23, 28], a potential risk factor for DDE [8, 9]. Therefore, we tested the direct and indirect effects, mediated by the preterm birth, of this chemokine in pregnant women on the occurrence of DDE-iu in their babies, adjusted to the socioeconomic conditions of the family and the child’s age. In this study, it was hypothesized that RANTES serum decrease contributes to the occurrence of DDE (Fig 1).

### Statistical analysis

The initial statistical analyzes were performed using the Stata® 14.0 software, estimating the absolute and percentage frequencies for categorical variables, as well as means (± standard deviations), medians, and 95% confidence intervals (95%CI). The comparison of frequencies between groups was performed by Fisher’s exact test or the Chi-squared test. The Student’s test or the Mann-Whitney U test was applied to compare the means or medians.

To investigate the effects (direct, indirect, and total) of RANTES on DDE in children aged 12.3 to 36 months old, the theoretical model was tested by SEM. This statistical analysis estimates a series of multiple regression equations. The model presupposes direct and indirect linear relations between a set of observed variables and constructs (latent variables). It consists of two sub-models: the measurement model, which establishes how the constructs are measured; and the structural model, which analyzes the theoretical model. The relationship was considered significant when the P-value of the standardized coefficient (SC) was <0.05 [32]. Negative SC indicates an inverse association and positive charges indicate a direct association [37]. We interpreted the SC of the structural model as follows: SC values close to 0.433 indicate a small effect, and above 0.749–0.839 indicate a major effect [32].

Latent variables are unobservable variables that reduce the measurement error of variables that are difficult to define or diagnose, minimizing measurement error in the process of estimation. A good latent variable has convergent validity, verified by SC greater than 0.50, but <0.95 (discriminant validity), indicating that each indicator measures different aspects of the construct [32].

In this analysis, the fit indices values considered to judge model adequacy were: p-value of the Chi-square (X2) for a Root Mean Square Error of Approximation (RMSEA) greater than 0.05; RMSEA less than 0.5; the upper limit of the 90% confidence interval of an RMSEA less than 0.08; Comparative Fit Index (CFI) and Tucker-Lewis Index (TLI) larger than 0.90; and Weighted Root Mean Square Residual (WRMR) less than 1.0 [32]. The SEM was performed using the software Mplus 7.0 (Muthen & Muthen, Philadelphia, Pennsylvania, United States).

## Results

The prevalence of DDE-iu was 7.95%, indicative of 26 children with at least one tooth affected. Regarding the type of DDE, the prevalence of opacity and hypoplasia was 4.89% (n = 16) and 3.67% (n = 12), respectively. The average age of the mother was 25.9 years (ranging from 14 to 45) and of the children was 16.37 months (ranging from 12.3 to 36).

Around thirty-six percent (36.39%) of pregnant women had semi-skilled manual jobs and 25.08% had unskilled labor positions. Most of the sample belonged to class C (65.14%) and had between nine and 11 years of school education (74.92%). The variables described in this table did not differ between groups (Table 1).

**Table 1:** Qualitative variables: Characterization of the population of the study, according to the incidence of DDE-iu BRISA (2011–2013).

Qualitative variables	Total (n = 327)		With DDE-iu (n = 26)		Without DDE-iu (n = 301)		P- value
	n	%	n	%	n	%	
Mother’s Occupation							0.650**
Unskilled labor	82	25.08	6	7.3	76	9.2	
Semi-skilled labor	119	36.39	10	8.4	109	91.6	
Skilled labor	18	5.5	0	0	18	100	
Office roles	58	17.74	5	8.6	53	91.4	
Higher-level	20	6.12	3	15.0	17	85.0	
Professional administrators / managers / directors / owners	7	2.14	1	14.3	6	85.7	
Other	23	7.03	1	4.35	22	95.65	
**Brazilian Economic Classification Criteria**							0.887**
A-B	53	16.11	4	7.55	49	92.45	
C	213	65.14	19	8.9	194	91.1	
D-E	44	13.46	2	4.55	42	94.55	
Other	17	5.20	1	94.1	16	5.9	
**Mother’s School Years**							0.619*
Up to 4 years	3	0.92	0	0	3	100.0	
5–8 years	36	11.01	1	2.8	35	97.2	
9–11 years	245	74.92	21	8.6	224	91.4	
12 or more years	42	12.84	4	9.5	38	90.5	
Other	1	0.31	0	0	1	100.0	
**Preterm Birth**							0.470**
Yes	109	33.3	7	26.92	102	33.89	
No	218	67.67	19	73.08	199	66.11	

Brazilian Economic Classification Criteria (Portuguese acronym: Associação Brasileira de Estudos e Pesquisas) DDE: Developmental Defect of enamel- Intrauterine N: absolute frequency; %: percentual;

*Fisher’s exact test

**Chi-squared test

The mean serum level of RANTES was 8,990.03 pg/mL, the mean child’s age was 16.37 months, and the mean income was R$ 1,431.37. The mean and median child’s age were higher in children with DDE when compared to the group without DDE (S1 File).

All model fit indicators were satisfactory. The Root Mean Square Error of Approximation (RMSEA) was less than 0.05. The upper limit of 90%CI was lower than 0.08. TLI and CFI indices showed values higher than 0.90, and the WRMR was lower than 1.00 (Table 2).

**Table 2:** Factorial load and p-value for verifying the validity of the construct and factorial structure of the model, standardized estimates.

Tuning indexes	Values- any DDE-iu	Values- Hypoplasia	Values- Opacity
N of free parameters	27	25	25
Degrees of freedom	17	17	17
χ^2†^	20.869	18.956	22.739
P-value of χ^2‡^	0.232	0.3311	0.1579
RMSEA^§^	0.029	0.029	0.050
IC 90% do RMSEA	0.000–0.065	0.000–0.086	0.000–0.099
CFI^¶^	0.964	0.969	0.902
TLI^††^	0.941	0.949	0.839
WRMR^‡‡^	0.609	0.610	0.679

^†^certifying Chi-square-χ2 (reference: lowest value).

^‡^Intervalo 90% confidence (reference: IC90% upper bound less than 0.08).

^§^ Root Mean Square Error of Approximation RMSEA–(reference: less than 0.05).

^¶^Comparative Fit Index–CFI (reference: greater than 0.90).

^††^Tucker Lewis Index-TFI (reference: greater than 0.90).

^‡‡^Weighted Root Mean Square Residual—WRMR (reference: less than 1.00).

The latent variable SES presented Standardized Factor Loadings (SFL) greater than 0.5, indicating good convergent validity, except mother’s education (SFL: 0.399), considering the DDE-iu outcome. The income variable was the only one to present an SFL lower than 0.5 (SFL: 0.477) when considering the outcome of hypoplasia (Table 3).

**Table 3:** Standardized factor loadings, standard errors, and P values for indicators of the latent variables.

**Latent Variable (any DDE-iu)**	**Standardized Factor Loadings**	**Standard Error**	***P*-Value**
*SES*			
Income	0.615	0.071	**<0.001**
BCEC	0.547	0.069	**<0.001**
Occupation	0.756	0.083	**<0.001**
Mother’s education	0.399	0.060	**<0.001**
**Latent Variable (Hypoplasia)**	**Standardized Factor Loadings**	**Standard Error**	***P*-Value**
*SES*			
Income	0.477	0.087	**<0.001**
BCEC	0.688	0.096	**<0.001**
Occupation	0.684	0.082	**<0.001**
Mother’s education	0.573	0.085	**<0.001**
**Latent Variable (Opacity)**	**Standardized Factor Loadings**	**Standard Error**	***P*-Value**
*SES*			
Income	0.500	0.087	**<0.001**
BCEC	0.639	0.092	**<0.001**
Occupation	0.722	0.083	**<0.001**
Mother’s education	0.563	0.086	**<0.001**

Based on the variables being explored in the study, there were no indirect effects of RANTES on the occurrence of DDE-iu (p>0.05). However, we could identify a significant direct effect. The lower serum levels of RANTES (SC: -0.137; p: 0.022) explain the higher occurrence of DDE-iu (SC: -0.137; p: 0.022) and occurrence of hypoplasia (SC: -0.190; p: 0.007). However, we did not identify an effect on the occurrence of opacity (S: 0.343; p: 0.074) (Table 4).

**Table 4:** Standardized estimates of total, direct, and indirect effects of the prediction model of serum RANTES in pregnancy on the incidence of Developmental Defects of Enamel (DDE-iu, hypoplasia, and opacity) BRISA (2011–2013).

Variables	Types of Effect	Any DDE-iu		Hypoplasia		Opacity	
		SC	P-Value	SC	P-Value	SC	P-Value
Effect of SES in DDE-iu	Total	0.057	0.452	0.104	0.173	0.023	0.281
	Indirect total	0.001	0.969	0.005	0.833	-0.005	0.882
	Indirect specific						
	SES→PB→DDE	0.007	0.541	0.000	0.961	-0.001	0.980
	SES→RANTES→DDE	-0.006	0.654	0.005	0.837	-0.005	0.868
	SES→RANTES→PB→DDE	<0.001	0.723	0.000	0.900	0.001	0.867
	Direct	0.056	0.448	0.099	0.203	0.868	0.468
Effect of Age in DDE-iu	Total	**0.113**	0.001*	-2.930	0.736	0.003	0.977
Effect of RANTES in DDE-iu	Total	**-0.153**	0.029*	**-0.185**	0.002*	0.303	0.102
	Indirect total	0.007	0.551	0.005	0.878	-0.040	0.422
	Indirect specific						
	RANTES→PB→DDE	-0.007	0.551	0.005	0.878	-0.040	0.422
	Direct	**-0.137**	0.022*	**-0.190**	0.007**	0.343	0.074

DDE-iu: Developmental Defect of Enamel-Intrauterine, PB: Preterm Birth, RANTES: Regulated on Activation Normal T Cell Expressed and Secreted, SC: Standardized Coefficient, SES: Socioeconomic Status

## Discussion

In this longitudinal study, including 327 mother-infant dyads, the lower serum levels of RANTES in pregnant women explain a higher occurrence of DDE-iu in the primary dentition of the children. The greater number of teeth with hypoplasia was associated with the lowest serum levels of RANTES. However, this association was not explained by preterm birth. The lower levels of RANTES did not explain the occurrence of the greater number of opacity lesions in pregnant women (S: 0.343; p: 0.074). However, this exposure was associated with a greater number of teeth with hypoplasia (SC: -0.190; p: 0.007). The lower prevalence of opacity in this population possibly contributed to the lower power to identify associations in this sample.

Lower levels of RANTES may influence the quantitative process of dental enamel deposition during amelogenesis, specifically in the secretion phase. For hypoplasia, a "minor effect" (-0.190) was observed. However, the sample size may have contributed to the decrease in this effect. In addition, even "minor effects" in hypoplasia can have major long-term clinical implications due to several issues ranging from aesthetics and sensitivity to a higher risk of caries and structural breakdown.

The association between RANTES and DDE-iu, measured in pregnant women during the 22^nd^ and 25^th^ weeks of pregnancy, has no precedent in the literature. The physiological action of RANTES is to attract and recruit CD4+ and CD8+ T lymphocytes and lymphocyte suppressor cells, and mediate immune tolerance [20]. It is known that the calcium ion–through transport and cellular signaling–plays a part in the processes of secretion and maturation of the enamel [38, 39]. Lower levels of RANTES in the blood during pregnancy may interfere with the process of amelogenesis teeth since this chemokine influences cellular depolarization induced by the prolonged influx of calcium (Ca++) and release of inflammatory cytokines that promote cell adhesion/aggregation [20, 22, 29].

RANTES is important for the fetus’ proper formation. A decrease in RANTES during pregnancy may result in disturbances in the apposition and maturation processes of amelogenesis [20]. Interestingly, decreases in RANTES have been associated with decreased levels of vitamin D [40], and vitamin D deficiency is considered a risk factor for increased enamel developmental defects [41].

The fact that RANTES was collected between the 22^nd^ and 25^th^ weeks of pregnancy is an important aspect of this study. DDE in babies may be influenced by stressful events that occurred during gestation, including the period in which this chemokine was measured in maternal blood. Additionally, the sixth week of pregnancy is when mineralization in the deciduous tooth germs is almost starting, but the period of formation and mineralization for enamel on the crown of deciduous teeth continues until six months (central incisor) to fourteen months (second molar) after birth [36]. This analysis may indicate a more accurate chronological order when investigating the association between RANTES, preterm birth, and DDE.

Our study found no association between RANTES and DDE mediated by preterm birth. The association between preterm birth and DDE is quite controversial. There are reports that preterm birth would increase the risk of DDE [8, 42], while other authors found no such association [10, 43]. The divergence of the results regarding the role of preterm birth in the occurrence of DDE can be partially explained by methodological differences in the study design, size, and criteria for the DDE classification, and the variables considered for the model adjustment. In a recent systematic review and meta-analysis, a greater risk of developing DDE was observed in preterm children, differing from the results of our manuscript [8]. However, this association can be explained by several factors. It is difficult to measure the GA based on the LMP informed by the woman, as this information can be influenced by memory bias. Confounding factors can interfere in the study of this association, as important variables are not always included to control confounding. In addition, the cross-sectional design, present in a considerable part of the works, is not the most suitable for studying etiological hypotheses; the case-control studies did not always present the appropriate pairing for the sample. In addition, the results of systematic reviews may be influenced by publication bias, and our study is a longitudinal follow-up, presents a robust statistical analysis, which is adequate to estimate associations for complex outcomes and with multifactorial etiology and has a good sample power; therefore it presents valid results.

The diagnosis of DDE is quite challenging, especially in the primary dentition, due to differences in eruption chronology, difficulties in performing dental examinations in children up to 36 months of age, and other pathologies that affect enamel formation, such as fluorosis and amelogenesis imperfecta. Differences in the methodology used for diagnosing DDE and, mainly, the large age group of the children included in the studies can distort the results. The presence of a separate incremental line (neonatal line) related to the time of birth allows distinguishing between prenatal and post-formed enamel, in addition to allowing the dating of these biological alterations [44, 45]; however, the chronology of tooth enamel formation was not considered in most investigations [10]. The diagnosis of DDE of intra or extrauterine origin is complex, and in an attempt to measure it, therefore we considered the uterine origin, all defects present in the occlusal/incisal third, based on the criterion proposed by Reid and Dean [36].

The convenience sample, the small sample size, and the non-inclusion of some predictor variables in the model (such as anemia and maternal nutritional deficiencies) were considered some limitations of this study. However, the sample presented sufficient power (85.29%) to identify the association between RANTES and DDE-iu. If, on the one hand, the origin of the sample in a university hospital in the capital of Maranhão, Brazil, reduces the external validity of the findings, on the other hand, it contributes to reducing the possibility of confounding bias, as this study sample was nested to BRISA cohort [33].

Some children still did not have all the deciduous teeth at 15 months. Studies carried out in Brazilian populations [46, 47] showed that tooth eruption is, in general, earlier in Brazilian children, especially in the Northeast region [46] (where the sample for our study was obtained from). Thus, most of the teeth had already erupted in our study sample, mainly the central incisors, lateral incisors, and first molars; these teeth have already been identified as the teeth with the highest incidence of DDE in previous studies [48, 49]. However, there are just as many reports in the literature that 2^nd^ molars are more affected by DDE [9, 50], including a Brazilian study (sample = 827 children) [50]. Therefore, we recognize the divergences in the literature, regarding the chronology of tooth eruption, prevalence, and distribution of teeth most affected by DDE and that the results indicated in these manuscripts should be interpreted with caution. Additionally, there are reports in the literature describing the hypomineralization of canines and deciduous 2^nd^ molars as occurring concomitantly, and these would be the teeth that would be mineralizing the incisal/occlusal 1/3 of the teeth at T0 (22^nd^-25^nd^ weeks), and could be the teeth that would not be present in the younger children who were examined as part of the study [13]. The inclusion of children aged between 12.3 and 36 months leads to some heterogeneity in terms of the number of primary teeth in the dental arch and also explains the prevalence of less than 10% in the sample of this study, but to minimize this difference and reduce possible confounding bias, we adjusted for the child’s age.

This study has strengths, such as the data collection in three moments. It is also a study design nested into a cohort, which is one of the most suitable for evaluating etiologic hypotheses. Also, the selection of DDE-iu as an outcome allows a more polished analysis of the effect of exposures during the gestational period. The outcome was regarded as a discrete variable, increasing the statistical power, whereas most investigations consider the outcome as a dichotomous variable. Finally, the way we collected the gestational age variable through LMP, combined with the ultrasound analysis, decreased a possible memory and measurement bias for the outcome.

Another strength of this study is the statistical method using SEM to simultaneously test the association of RANTES and DDE-iu by exploring direct and indirect pathways. By being able to estimate a series of separate and interdependent multiple regression equations, this method tends to yield more reliable results. Moreover, it allows the estimate of the total, direct, and indirect effects between variables, presenting the ones mediating the total effect [32]. Also, this method yields results that are easy to interpret and allows us to work with initial losses of variables that can be imputed by the method of estimation. The construction of the latent variables for the SES also reduced possible measurement errors.

## Conclusion

In this study, DDE in the primary dentition was explained by the reduction in serum levels of the chemokine RANTES in pregnant women at 22 to 25 weeks of GA. The effect of the low serum RANTES levels occurred on hypoplasia-like defects but not on opacity. The maternal immune response can be considered a potentially relevant factor for the etiology of DDE in deciduous dentition, pointing to the need for greater control of women’s health during pregnancy to reduce developmental defects of enamel. However, more evidence supported by clinical, laboratory, and epidemiological studies is still needed.

## Supporting information

S1 File Quantitative variables: Characterization of the population of the study, according to the incidence of DDE-iu BRISA (2011–2013). (DOCX)

S2 File (OUT)

S3 File (PDF)

S4 File (OUT)

S5 File (PDF)

S6 File (OUT)

S7 File (PDF)
