# Supplementary material for: RANTES and developmental defects of enamel in children: A Brazilian prenatal cohort (BRISA)
Source: PLoS One. 2023 Jul 27;18(7):e0284606. doi: 10.1371/journal.pone.0284606 (PMC10374131; doi:10.1371/journal.pone.0284606)
Supplement: S1 File — (DOCX) [file pone.0284606.s001.docx]

**Supl 1.** Quantitative variables: Characterization of the population of the study, according to the incidence of DDE-iu BRISA (2011-2013)

#

| **Quantitative variables** | **Total** | | | | **With DDE-iu** | | | | **Without DDE-iu** | | | | **P-value** |
| --- | --- | --- | --- | --- | --- | --- | --- | --- | --- | --- | --- | --- | --- |
|  | 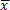 | **SD** | **Md** | **95%CI** | 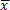 | **SD** | **Md** | **95%CI** | 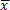 | **SD** | **Md** | **95%CI** |  |
| Income (R$) | 1,431.37 | 2,863.40 | 1,210 | 1,119.86 – 1,742.88 | 1,777.5 | 1,432.9 | 1,255 | 1,198.73 – 2,356.26 | 1,401.47 | 2,954.19 | 1,255 | 1,066.38 – 1,736.56 | 0.260* |
| Child’s age | 16.37 | 2.63 | 15.63 | 16.09 -16.67 | 19.34 | 5.77 | 16.42 | 17 - 21.67 | 16.13 | 1.98 | 15.59 | 15.90 - 16.35 | **<000.1**** |
| RANTES (pg/mL) | 8,990.03 | 6,512.67 | 7,489 | 8,145.16 – 9,835.24 | 8,810.94 | 6,413.86 | 7,148 | 7,940.51 – 9,681.38 | 10,791.31 | 7,564.13 | 1,1624 | 7,348.15 – 14,234.46 | 0.092* |

- : mean; SD: standard deviation; Md: median; Statistically significant differences (P < 0.05) are in bold *Mann-Whitney U Test **Student’s t-test
