# Supplementary material for: RANTES and developmental defects of enamel in children: A Brazilian prenatal cohort (BRISA)
Source: PLoS One. 2023 Jul 27;18(7):e0284606. doi: 10.1371/journal.pone.0284606 (PMC10374131; doi:10.1371/journal.pone.0284606)
Supplement: S3 File — (PDF) [file pone.0284606.s003.pdf]

```
Mplus VERSION 7.3
MUTHEN & MUTHEN
07/29/2022   2:21 PM

INPUT INSTRUCTIONS

  TITLE: ANALISE DE ELISA - ARTIGO desfechos

    DATA:
        File is rantes_zz4_27-11-17.dat;

    Variable:
        NAMES are bpn e g k o q r s t v ab q2 opac hipoplasia dde1 dde2
b2_imp ib
        c10 c11 c10c c11c zz4 hip outros outros2;
        USEVARIABLES are e q2 r s t hip b2_imp c10c;
        CATEGORICAL are e s c10c;
        MISSING ARE ALL (-9999);

    ANALYSIS: PARAMETERIZATION = THETA;

    MODEL:
        ses BY q2 r s t;
        c10c ON ses;
        e ON c10c ses;
        hip ON b2_imp e c10c ses;

    MODEL INDIRECT:
        hip IND ses;
        hip IND c10c;

    OUTPUT: MODINDICES RESIDUAL STDYX;



*** WARNING
  Data set contains cases with missing on x-variables.
  These cases were not included in the analysis.
  Number of cases with missing on x-variables:  30
   1 WARNING(S) FOUND IN THE INPUT INSTRUCTIONS



ANALISE DE ELISA - ARTIGO desfechos

SUMMARY OF ANALYSIS

Number of groups                                                 1
Number of observations                                         135

Number of dependent variables                                    7
Number of independent variables                                  1
Number of continuous latent variables                            1
```

Observed dependent variables

Continuous
Q2 R T HIP

Binary and ordered categorical (ordinal)
E S C10C

Observed independent variables
B2_IMP

Continuous latent variables
SES

Estimator	WLSMV
Maximum number of iterations	1000
Convergence criterion	0.500D-04
Maximum number of steepest descent iterations	20
Maximum number of iterations for H1	2000
Convergence criterion for H1	0.100D-03
Parameterization	THETA

Input data file(s)
rantes_zz4_27-11-17.dat

Input data format FREE

SUMMARY OF DATA

Number of missing data patterns 10

COVARIANCE COVERAGE OF DATA

Minimum covariance coverage value 0.100

PROPORTION OF DATA PRESENT

Covariance Coverage

	E	Q2	R	S	T
E	0.978				
Q2	0.941	0.963			
R	0.904	0.896	0.926		
S	0.948	0.941	0.911	0.970	
T	0.978	0.963	0.926	0.970	1.000
HIP	0.956	0.941	0.911	0.948	0.978

```
 C10C           0.970         0.956         0.919         0.963
0.993


           Covariance Coverage
              HIP           C10C
              ________      ________
 HIP            0.978
 C10C           0.970         0.993


UNIVARIATE PROPORTIONS AND COUNTS FOR CATEGORICAL VARIABLES

    E
      Category 1    0.689       91.000
      Category 2    0.083       11.000
      Category 3    0.076       10.000
      Category 4    0.152       20.000
    S
      Category 1    0.153       20.000
      Category 2    0.664       87.000
      Category 3    0.183       24.000
    C10C
      Category 1    0.575       77.000
      Category 2    0.425       57.000



THE MODEL ESTIMATION TERMINATED NORMALLY



MODEL FIT INFORMATION

Number of Free Parameters                       25

Chi-Square Test of Model Fit

          Value                              18.956*
          Degrees of Freedom                    17
          P-Value                            0.3311

*   The chi-square value for MLM, MLMV, MLR, ULSMV, WLSM and WLSMV cannot
be used
    for chi-square difference testing in the regular way.  MLM, MLR and
WLSM
    chi-square difference testing is described on the Mplus website.
MLMV, WLSMV,
    and ULSMV difference testing is done using the DIFFTEST option.

RMSEA (Root Mean Square Error Of Approximation)

          Estimate                            0.029
          90 Percent C.I.                     0.000  0.086
```

```
          Probability RMSEA <= .05           0.663

CFI/TLI

          CFI                                0.969
          TLI                                0.949

Chi-Square Test of Model Fit for the Baseline Model

          Value                             91.620
          Degrees of Freedom                    28
          P-Value                           0.0000

WRMR (Weighted Root Mean Square Residual)

          Value                              0.610



MODEL RESULTS

                                                    Two-Tailed
                    Estimate       S.E.  Est./S.E.    P-Value

 SES      BY
    Q2                 1.000      0.000    999.000    999.000
    R                  0.683      0.221      3.085      0.002
    S                  0.707      0.192      3.689      0.000
    T                  0.234      0.065      3.618      0.000

 C10C     ON
    SES               -0.020      0.098     -0.207      0.836

 E        ON
    SES                0.005      0.099      0.051      0.959

 HIP      ON
    SES                1.855      1.515      1.224      0.221

 E        ON
    C10C               0.189      0.141      1.336      0.182

 HIP      ON
    B2_IMP            -2.930      8.699     -0.337      0.736
    E                  0.596      3.821      0.156      0.876
    C10C              -4.720      1.778     -2.654      0.008

 Intercepts
    Q2                 4.761      0.278     17.148      0.000
    R                  2.529      0.155     16.322      0.000
    T                  2.012      0.056     35.958      0.000
    HIP                7.731      3.112      2.485      0.013

 Thresholds
```

E$1	0.549	0.132	4.165	0.000
E$2	0.808	0.138	5.840	0.000
E$3	1.096	0.149	7.361	0.000
S$1	-1.376	0.215	-6.394	0.000
S$2	1.271	0.228	5.576	0.000
C10C$1	0.252	0.123	2.053	0.040
**Variances**				
SES	1.762	0.874	2.017	0.044
**Residual Variances**				
Q2	5.992	1.167	5.135	0.000
R	0.914	0.215	4.251	0.000
T	0.197	0.032	6.222	0.000
HIP	587.432	44.040	13.339	0.000

STANDARDIZED MODEL RESULTS

STDYX Standardization

	Estimate	S.E.	Est./S.E.	Two-Tailed P-Value
**SES BY**				
Q2	0.477	0.087	5.453	0.000
R	0.688	0.096	7.147	0.000
S	0.684	0.082	8.379	0.000
T	0.573	0.085	6.721	0.000
**C10C ON**				
SES	-0.027	0.131	-0.205	0.837
**E ON**				
SES	0.007	0.130	0.051	0.959
**HIP ON**				
SES	0.099	0.078	1.273	0.203
**E ON**				
C10C	0.186	0.134	1.383	0.167
**HIP ON**				
B2_IMP	-0.071	0.209	-0.338	0.735
E	0.024	0.156	0.156	0.876
C10C	-0.190	0.070	-2.696	0.007
**Intercepts**				
Q2	1.710	0.204	8.377	0.000
R	1.920	0.259	7.408	0.000
T	3.717	0.187	19.905	0.000
HIP	0.311	0.133	2.342	0.019

**Thresholds**				
E$1	0.539	0.129	4.188	0.000
E$2	0.794	0.135	5.887	0.000
E$3	1.077	0.145	7.433	0.000
S$1	-1.003	0.141	-7.137	0.000
S$2	0.927	0.137	6.777	0.000
C10C$1	0.252	0.123	2.053	0.040
**Variances**				
SES	1.000	0.000	999.000	999.000
**Residual Variances**				
Q2	0.773	0.083	9.272	0.000
R	0.527	0.132	3.977	0.000
T	0.671	0.098	6.868	0.000
HIP	0.949	0.037	25.941	0.000

R-SQUARE

Observed Variable	Estimate	S.E.	Est./S.E.	Two-Tailed P-Value	Scale Factors
E	0.034	0.050	0.691	0.489	0.983
Q2	0.227	0.083	2.727	0.006	
R	0.473	0.132	3.574	0.000	
S	0.468	0.112	4.189	0.000	0.729
T	0.329	0.098	3.361	0.001	
HIP	0.051	0.037	1.385	0.166	
C10C	0.001	0.007	0.103	0.918	1.000

QUALITY OF NUMERICAL RESULTS

Condition Number for the Information Matrix 0.111E-04
(ratio of smallest to largest eigenvalue)

TOTAL, TOTAL INDIRECT, SPECIFIC INDIRECT, AND DIRECT EFFECTS

	Estimate	S.E.	Est./S.E.	Two-Tailed P-Value
**Effects from SES to HIP**				
Total	1.951	1.468	1.329	0.184
Total indirect	0.096	0.453	0.212	0.832
Specific indirect				
HIP				
E				
SES	0.003	0.062	0.049	0.961

```
    HIP
    C10C
    SES                 0.095      0.459      0.208      0.835

    HIP
    E
    C10C
    SES                -0.002      0.018     -0.127      0.899

  Direct
    HIP
    SES                 1.855      1.515      1.224      0.221


Effects from C10C to HIP

  Total                -4.607      1.521     -3.030      0.002
  Total indirect        0.113      0.732      0.154      0.878

  Specific indirect

    HIP
    E
    C10C                0.113      0.732      0.154      0.878

  Direct
    HIP
    C10C               -4.720      1.778     -2.654      0.008


STANDARDIZED TOTAL, TOTAL INDIRECT, SPECIFIC INDIRECT, AND DIRECT EFFECTS


STDYX Standardization

                                                    Two-Tailed
                    Estimate       S.E.  Est./S.E.    P-Value

Effects from SES to HIP

  Total                 0.104      0.076      1.363      0.173
  Total indirect        0.005      0.024      0.210      0.833

  Specific indirect

    HIP
    E
    SES                 0.000      0.003      0.049      0.961

    HIP
    C10C
    SES                 0.005      0.025      0.206      0.837
```

```
    HIP
    E
    C10C
    SES                0.000      0.001     -0.126      0.900

  Direct
    HIP
    SES                0.099      0.078      1.273      0.203


Effects from C10C to HIP

  Total               -0.185      0.060     -3.095      0.002
  Total indirect       0.005      0.029      0.154      0.878

  Specific indirect

    HIP
    E
    C10C               0.005      0.029      0.154      0.878

  Direct
    HIP
    C10C              -0.190      0.070     -2.696      0.007



RESIDUAL OUTPUT


     ESTIMATED MODEL AND RESIDUALS (OBSERVED - ESTIMATED)


           Model Estimated Means/Intercepts/Thresholds
              E$1           E$2           E$3           Q2            R
              ________      ________      ________      ________
________
      1         0.539         0.794         1.077         4.761
2.529


           Model Estimated Means/Intercepts/Thresholds
              S$1           S$2           T             HIP
C10C$1
              ________      ________      ________      ________
________
      1        -1.003         0.927         2.012         7.731
0.252


           Residuals for Means/Intercepts/Thresholds
              E$1           E$2           E$3           Q2            R
              ________      ________      ________      ________
________
```

1	0.000	0.000	0.000	0.000	0.000

Residuals for Means/Intercepts/Thresholds

	S$1	S$2	T	HIP	C10C$1
1	0.000	0.000	0.000	0.000	0.000

Model Estimated Slopes

	B2_IMP
E	0.000
Q2	0.000
R	0.000
S	0.000
T	0.000
HIP	-2.930
C10C	0.000

Residuals for Slopes

	B2_IMP
E	0.145
Q2	0.176
R	-0.347
S	0.074
T	-0.092
HIP	-0.001
C10C	0.210

Model Estimated Covariances/Correlations/Residual Correlations

	E	Q2	R	S	T
E					
Q2	0.002	7.755			
R	0.001	1.203	1.736		
S	0.001	0.908	0.620		
T	0.001	0.412	0.281	0.212	0.293
HIP	-0.266	3.438	2.347	1.772	0.804
C10C	0.186	-0.036	-0.024	-0.018	-0.008

Model Estimated Covariances/Correlations/Residual Correlations

	HIP	C10C
HIP	615.719	
C10C	-4.675	

Residuals for Covariances/Correlations/Residual Correlations

	E	Q2	R	S	T
E					
Q2	-0.339	0.000			
R	-0.012	-0.268	0.000		
S	0.023	0.410	-0.032		
T	0.065	-0.038	0.069	-0.031	0.000
HIP	-0.002	-3.887	0.545	1.738	0.082
C10C	0.000	0.200	-0.051	0.080	-0.044

Residuals for Covariances/Correlations/Residual Correlations

	HIP	C10C
HIP	0.002	
C10C	0.000	

UNIVARIATE PROPORTIONS FOR CATEGORICAL VARIABLES

	Observed	Estimated	Residual (Observed-Estimated)
E			
Category 1	0.689	0.705	-0.016
Category 2	0.083	0.081	0.002
Category 3	0.076	0.073	0.003
Category 4	0.152	0.141	0.011
S			
Category 1	0.153	0.158	-0.005
Category 2	0.664	0.665	-0.001
Category 3	0.183	0.177	0.006
C10C			
Category 1	0.575	0.599	-0.025
Category 2	0.425	0.401	0.025

MODEL MODIFICATION INDICES

NOTE: Modification indices for direct effects of observed dependent variables
regressed on covariates and residual covariances among observed dependent

variables may not be included. To include these, request MODINDICES
(ALL).

Minimum M.I. value for printing the modification index   10.000

                                   M.I.   E.P.C. Std E.P.C. StdYX
E.P.C.

No modification indices above the minimum value.

DIAGRAM INFORMATION

Use View Diagram under the Diagram menu in the Mplus Editor to view the diagram.
If running Mplus from the Mplus Diagrammer, the diagram opens automatically.

Diagram output

e:\@erika_thomaz\@a_ufma\a_orientacoes\elisa_miranda\mestrado\artigo_rantes\banco_plos_29-07-22h

Beginning Time: 14:21:37
Ending Time: 14:21:38
Elapsed Time: 00:00:01

MUTHEN & MUTHEN
3463 Stoner Ave.
Los Angeles, CA 90066

Tel: (310) 391-9971
Fax: (310) 391-8971
Web: www.StatModel.com
Support: Support@StatModel.com

Copyright (c) 1998-2014 Muthen & Muthen
