# Supplementary material for: RANTES and developmental defects of enamel in children: A Brazilian prenatal cohort (BRISA)
Source: PLoS One. 2023 Jul 27;18(7):e0284606. doi: 10.1371/journal.pone.0284606 (PMC10374131; doi:10.1371/journal.pone.0284606)
Supplement: S5 File — (PDF) [file pone.0284606.s005.pdf]

```
Mplus VERSION 7.3
MUTHEN & MUTHEN
07/29/2022   2:18 PM

INPUT INSTRUCTIONS

  TITLE: ANALISE DE ELISA - ARTIGO desfechos

    DATA:
        File is rantes_zz4_27-11-17.dat;

    Variable:
        NAMES are bpn e g k o q r s t v ab q2 opac hipoplasia dde1 dde2
b2_imp ib
        c10 c11 c10c c11c zz4 hip outros outros2;
        USEVARIABLES are e q2 r s t opac b2_imp c10c;
        CATEGORICAL are e s c10c;
        MISSING ARE ALL (-9999);

    ANALYSIS: PARAMETERIZATION = THETA;

    MODEL:
        ses BY q2 r s t;
        c10c ON ses;
        e ON c10c ses;
        opac ON b2_imp e c10c ses;

    MODEL INDIRECT:
        opac IND ses;
        opac IND c10c;

    OUTPUT: MODINDICES RESIDUAL STDYX;



*** WARNING
  Data set contains cases with missing on x-variables.
  These cases were not included in the analysis.
  Number of cases with missing on x-variables:  30
   1 WARNING(S) FOUND IN THE INPUT INSTRUCTIONS



ANALISE DE ELISA - ARTIGO desfechos

SUMMARY OF ANALYSIS

Number of groups                                                 1
Number of observations                                         135

Number of dependent variables                                    7
Number of independent variables                                  1
Number of continuous latent variables                            1
```

Observed dependent variables

Continuous
Q2 R T OPAC

Binary and ordered categorical (ordinal)
E S C10C

Observed independent variables
B2_IMP

Continuous latent variables
SES

Estimator	WLSMV
Maximum number of iterations	1000
Convergence criterion	0.500D-04
Maximum number of steepest descent iterations	20
Maximum number of iterations for H1	2000
Convergence criterion for H1	0.100D-03
Parameterization	THETA

Input data file(s)
rantes_zz4_27-11-17.dat

Input data format FREE

SUMMARY OF DATA

Number of missing data patterns 8

COVARIANCE COVERAGE OF DATA

Minimum covariance coverage value 0.100

PROPORTION OF DATA PRESENT

Covariance Coverage

	E	Q2	R	S	T
E	0.978				
Q2	0.941	0.963			
R	0.904	0.896	0.926		
S	0.948	0.941	0.911	0.970	
T	0.978	0.963	0.926	0.970	1.000
OPAC	0.978	0.963	0.926	0.970	1.000

C10C	0.970	0.956	0.919	0.963	0.993

Covariance Coverage

	OPAC	C10C
OPAC	1.000	
C10C	0.993	0.993

UNIVARIATE PROPORTIONS AND COUNTS FOR CATEGORICAL VARIABLES

E		
Category 1	0.689	91.000
Category 2	0.083	11.000
Category 3	0.076	10.000
Category 4	0.152	20.000
S		
Category 1	0.153	20.000
Category 2	0.664	87.000
Category 3	0.183	24.000
C10C		
Category 1	0.575	77.000
Category 2	0.425	57.000

THE MODEL ESTIMATION TERMINATED NORMALLY

MODEL FIT INFORMATION

Number of Free Parameters 25

Chi-Square Test of Model Fit

Value	22.739*
Degrees of Freedom	17
P-Value	0.1579

* The chi-square value for MLM, MLMV, MLR, ULSMV, WLSM and WLSMV cannot be used for chi-square difference testing in the regular way. MLM, MLR and WLSM chi-square difference testing is described on the Mplus website. MLMV, WLSMV, and ULSMV difference testing is done using the DIFFTEST option.

RMSEA (Root Mean Square Error Of Approximation)

Estimate	0.050	
90 Percent C.I.	0.000	0.099

```
          Probability RMSEA <= .05           0.457

CFI/TLI

          CFI                                0.902
          TLI                                0.839

Chi-Square Test of Model Fit for the Baseline Model

          Value                             86.777
          Degrees of Freedom                    28
          P-Value                           0.0000

WRMR (Weighted Root Mean Square Residual)

          Value                              0.679



MODEL RESULTS

                                                    Two-Tailed
                    Estimate       S.E.  Est./S.E.    P-Value

 SES      BY
    Q2                 1.000      0.000    999.000    999.000
    R                  0.604      0.191      3.171      0.002
    S                  0.749      0.217      3.455      0.001
    T                  0.219      0.061      3.597      0.000

 C10C     ON
    SES               -0.016      0.094     -0.169      0.866

 E        ON
    SES                0.002      0.095      0.025      0.980

 OPAC     ON
    SES                0.028      0.039      0.725      0.468

 E        ON
    C10C               0.189      0.141      1.335      0.182

 OPAC     ON
    B2_IMP             0.003      0.109      0.028      0.977
    E                 -0.212      0.192     -1.102      0.271
    C10C               0.343      0.192      1.789      0.074

 Intercepts
    Q2                 4.761      0.278     17.148      0.000
    R                  2.529      0.155     16.322      0.000
    T                  2.012      0.056     35.958      0.000
    OPAC               0.095      0.170      0.562      0.574

 Thresholds
```

```
    E$1                0.549      0.132      4.165      0.000
    E$2                0.808      0.138      5.840      0.000
    E$3                1.096      0.149      7.361      0.000
    S$1               -1.450      0.238     -6.084      0.000
    S$2                1.339      0.268      4.990      0.000
    C10C$1             0.252      0.123      2.053      0.040

 Variances
    SES                1.939      0.932      2.079      0.038

 Residual Variances
    Q2                 5.816      1.127      5.162      0.000
    R                  1.027      0.201      5.105      0.000
    T                  0.200      0.032      6.247      0.000
    OPAC               0.216      0.119      1.821      0.069


STANDARDIZED MODEL RESULTS


STDYX Standardization

                                                    Two-Tailed
                    Estimate       S.E.  Est./S.E.    P-Value

 SES      BY
    Q2                 0.500      0.087      5.747      0.000
    R                  0.639      0.092      6.979      0.000
    S                  0.722      0.083      8.710      0.000
    T                  0.563      0.086      6.535      0.000

 C10C     ON
    SES               -0.022      0.131     -0.168      0.867

 E        ON
    SES                0.003      0.130      0.025      0.980

 OPAC     ON
    SES                0.066      0.091      0.727      0.467

 E        ON
    C10C               0.186      0.134      1.383      0.167

 OPAC     ON
    B2_IMP             0.003      0.110      0.028      0.977
    E                 -0.362      0.329     -1.102      0.271
    C10C               0.578      0.305      1.891      0.059

 Intercepts
    Q2                 1.710      0.204      8.377      0.000
    R                  1.920      0.259      7.408      0.000
    T                  3.717      0.187     19.905      0.000
    OPAC               0.160      0.290      0.553      0.580
```

	Estimate	S.E.	Est./S.E.	Two-Tailed P-Value
**Thresholds**				
E$1	0.539	0.129	4.188	0.000
E$2	0.794	0.135	5.887	0.000
E$3	1.077	0.145	7.433	0.000
S$1	-1.003	0.141	-7.137	0.000
S$2	0.927	0.137	6.777	0.000
C10C$1	0.252	0.123	2.053	0.040
**Variances**				
SES	1.000	0.000	999.000	999.000
**Residual Variances**				
Q2	0.750	0.087	8.621	0.000
R	0.592	0.117	5.063	0.000
T	0.683	0.097	7.032	0.000
OPAC	0.610	0.368	1.659	0.097

R-SQUARE

Observed Variable	Estimate	S.E.	Est./S.E.	Two-Tailed P-Value	Scale Factors
E	0.034	0.050	0.691	0.489	0.983
Q2	0.250	0.087	2.874	0.004	
R	0.408	0.117	3.490	0.000	
S	0.521	0.120	4.355	0.000	0.692
T	0.317	0.097	3.267	0.001	
OPAC	0.390	0.368	1.060	0.289	
C10C	0.000	0.006	0.084	0.933	1.000

QUALITY OF NUMERICAL RESULTS

Condition Number for the Information Matrix 0.263E-03
(ratio of smallest to largest eigenvalue)

TOTAL, TOTAL INDIRECT, SPECIFIC INDIRECT, AND DIRECT EFFECTS

	Estimate	S.E.	Est./S.E.	Two-Tailed P-Value
**Effects from SES to OPAC**				
Total	0.023	0.021	1.079	0.281
Total indirect	-0.005	0.036	-0.148	0.882
Specific indirect				
OPAC				
E				
SES	-0.001	0.020	-0.025	0.980

```
    OPAC
    C10C
    SES                 -0.005      0.033     -0.166      0.868

    OPAC
    E
    C10C
    SES                  0.001      0.004      0.167      0.867

  Direct
    OPAC
    SES                  0.028      0.039      0.725      0.468


Effects from C10C to OPAC

  Total                  0.303      0.185      1.636      0.102
  Total indirect        -0.040      0.050     -0.803      0.422

  Specific indirect

    OPAC
    E
    C10C                -0.040      0.050     -0.803      0.422

  Direct
    OPAC
    C10C                 0.343      0.192      1.789      0.074


STANDARDIZED TOTAL, TOTAL INDIRECT, SPECIFIC INDIRECT, AND DIRECT EFFECTS


STDYX Standardization

                                                        Two-Tailed
                    Estimate       S.E.  Est./S.E.    P-Value

Effects from SES to OPAC

  Total                  0.053      0.047      1.142      0.253
  Total indirect        -0.012      0.084     -0.147      0.883

  Specific indirect

    OPAC
    E
    SES                 -0.001      0.047     -0.025      0.980

    OPAC
    C10C
    SES                 -0.013      0.077     -0.165      0.869
```

```
    OPAC
   E
    C10C
    SES                  0.001      0.009      0.166      0.868

  Direct
    OPAC
    SES                  0.066      0.091      0.727      0.467


Effects from C10C to OPAC

  Total                  0.510      0.296      1.723      0.085
  Total indirect        -0.067      0.084     -0.804      0.421

  Specific indirect

    OPAC
    E
    C10C                -0.067      0.084     -0.804      0.421

  Direct
    OPAC
    C10C                 0.578      0.305      1.891      0.059


RESIDUAL OUTPUT


     ESTIMATED MODEL AND RESIDUALS (OBSERVED - ESTIMATED)


           Model Estimated Means/Intercepts/Thresholds
              E$1           E$2           E$3           Q2            R
              ________      ________      ________      ________
________
      1         0.539         0.794         1.077         4.761
2.529


           Model Estimated Means/Intercepts/Thresholds
              S$1           S$2           T             OPAC
C10C$1
              ________      ________      ________      ________
________
      1        -1.003         0.927         2.012         0.095
0.252


           Residuals for Means/Intercepts/Thresholds
              E$1           E$2           E$3           Q2            R
              ________      ________      ________      ________
________
```

1	0.000	0.000	0.000	0.000	0.000

Residuals for Means/Intercepts/Thresholds

	S$1	S$2	T	OPAC	C10C$1
1	0.000	0.000	0.000	0.000	0.000

Model Estimated Slopes

	B2_IMP
E	0.000
Q2	0.000
R	0.000
S	0.000
T	0.000
OPAC	0.003
C10C	0.000

Residuals for Slopes

	B2_IMP
E	0.145
Q2	0.176
R	-0.347
S	0.074
T	-0.092
OPAC	0.000
C10C	0.210

Model Estimated Covariances/Correlations/Residual Correlations

	E	Q2	R	S	T
E					
Q2	-0.001	7.755			
R	-0.001	1.172	1.736		
S	-0.001	1.005	0.607		
T	0.000	0.425	0.257	0.220	0.293
OPAC	-0.152	0.044	0.027	0.023	0.010
C10C	0.186	-0.031	-0.019	-0.016	-0.007

Model Estimated Covariances/Correlations/Residual Correlations

	OPAC	C10C
OPAC	0.354	
C10C	0.303	

Residuals for Covariances/Correlations/Residual Correlations

	E	Q2	R	S	T
E					
Q2	-0.335	0.000			
R	-0.010	-0.236	0.000		
S	0.024	0.314	-0.019		
T	0.066	-0.050	0.093	-0.039	0.000
OPAC	0.000	-0.078	-0.053	0.031	0.007
C10C	0.000	0.195	-0.056	0.077	-0.046

Residuals for Covariances/Correlations/Residual Correlations

	OPAC	C10C
OPAC	0.000	
C10C	0.000	

UNIVARIATE PROPORTIONS FOR CATEGORICAL VARIABLES

	Observed	Estimated	Residual (Observed-Estimated)
E			
Category 1	0.689	0.705	-0.016
Category 2	0.083	0.081	0.002
Category 3	0.076	0.073	0.003
Category 4	0.152	0.141	0.011
S			
Category 1	0.153	0.158	-0.005
Category 2	0.664	0.665	-0.001
Category 3	0.183	0.177	0.006
C10C			
Category 1	0.575	0.599	-0.025
Category 2	0.425	0.401	0.025

MODEL MODIFICATION INDICES

NOTE: Modification indices for direct effects of observed dependent variables
regressed on covariates and residual covariances among observed dependent

variables may not be included. To include these, request MODINDICES (ALL).

Minimum M.I. value for printing the modification index 10.000

M.I. E.P.C. Std E.P.C. StdYX E.P.C.

No modification indices above the minimum value.

DIAGRAM INFORMATION

Use View Diagram under the Diagram menu in the Mplus Editor to view the diagram.
If running Mplus from the Mplus Diagrammer, the diagram opens automatically.

Diagram output

e:\@erika_thomaz\@a_ufma\a_orientacoes\elisa_miranda\mestrado\artigo_rantes\banco_plos_29-07-22o

Beginning Time: 14:18:37
Ending Time: 14:18:37
Elapsed Time: 00:00:00

MUTHEN & MUTHEN
3463 Stoner Ave.
Los Angeles, CA 90066

Tel: (310) 391-9971
Fax: (310) 391-8971
Web: www.StatModel.com
Support: Support@StatModel.com

Copyright (c) 1998-2014 Muthen & Muthen
