# Supplementary material for: RANTES and developmental defects of enamel in children: A Brazilian prenatal cohort (BRISA)
Source: PLoS One. 2023 Jul 27;18(7):e0284606. doi: 10.1371/journal.pone.0284606 (PMC10374131; doi:10.1371/journal.pone.0284606)
Supplement: S7 File — (PDF) [file pone.0284606.s007.pdf]

```
Mplus VERSION 7.3
MUTHEN & MUTHEN
07/29/2022   2:06 PM

INPUT INSTRUCTIONS

  TITLE: ANALISE DE ELISA - ARTIGO desfechos

    DATA:
        File is rantes_zz4_27-11-17.dat;

    Variable:
        NAMES are bpn e g k o q r s t v ab q2 opac dde1 dde2 b2_imp ib
c10 c11 c10c c11c zz4
        USEVARIABLES are e q2 r s t zz4 b2_imp c10c;
        CATEGORICAL are e s c10c;
        MISSING ARE ALL (-9999);

    ANALYSIS: PARAMETERIZATION = THETA;

    MODEL:
        ses BY q2 r s t;
        c10c ON ses;
        e ON c10c ses;
        zz4 ON b2_imp e c10c ses;

    MODEL INDIRECT:
        zz4 IND ses;
        zz4 IND c10c;

    OUTPUT: MODINDICES RESIDUAL STDYX;



*** WARNING
  Input line exceeded 90 characters. Some input may be truncated.
        NAMES are bpn e g k o q r s t v ab q2 opac dde1 dde2 b2_imp ib
c10 c11 c10c c11c zz4;
   1 WARNING(S) FOUND IN THE INPUT INSTRUCTIONS



ANALISE DE ELISA - ARTIGO desfechos

SUMMARY OF ANALYSIS

Number of groups                                                 1
Number of observations                                         270

Number of dependent variables                                    7
Number of independent variables                                  1
Number of continuous latent variables                            1

Observed dependent variables
```

```
  Continuous
   Q2          R           T           ZZ4

  Binary and ordered categorical (ordinal)
   E           S           C10C

Observed independent variables
   B2_IMP

Continuous latent variables
   SES


Estimator                                                    WLSMV
Maximum number of iterations                                  1000
Convergence criterion                                    0.500D-04
Maximum number of steepest descent iterations                   20
Maximum number of iterations for H1                           2000
Convergence criterion for H1                             0.100D-03
Parameterization                                             THETA

Input data file(s)
  rantes_zz4_27-11-17.dat

Input data format  FREE


SUMMARY OF DATA

     Number of missing data patterns            11


COVARIANCE COVERAGE OF DATA

Minimum covariance coverage value   0.100


     PROPORTION OF DATA PRESENT


           Covariance Coverage
              E             Q2            R             S             T
              ________      ________      ________      ________
________
 E             0.981
 Q2            0.952         0.970
 R             0.922         0.919         0.941
 S             0.933         0.933         0.907         0.952
 T             0.981         0.967         0.937         0.948
0.996
 ZZ4           0.974         0.963         0.933         0.944
0.989
```

```
 C10C           0.981         0.970         0.941         0.952
0.996


           Covariance Coverage
              ZZ4           C10C
              ________      ________
 ZZ4            0.993
 C10C           0.993         1.000


UNIVARIATE PROPORTIONS AND COUNTS FOR CATEGORICAL VARIABLES

    E
      Category 1    0.713      189.000
      Category 2    0.060       16.000
      Category 3    0.057       15.000
      Category 4    0.170       45.000
    S
      Category 1    0.136       35.000
      Category 2    0.704      181.000
      Category 3    0.160       41.000
    C10C
      Category 1    0.600      162.000
      Category 2    0.389      105.000
      Category 3    0.007        2.000
      Category 4    0.004        1.000



THE MODEL ESTIMATION TERMINATED NORMALLY



MODEL FIT INFORMATION

Number of Free Parameters                       27

Chi-Square Test of Model Fit

          Value                             20.869*
          Degrees of Freedom                    17
          P-Value                           0.2322

*   The chi-square value for MLM, MLMV, MLR, ULSMV, WLSM and WLSMV cannot
be used
    for chi-square difference testing in the regular way.  MLM, MLR and
WLSM
    chi-square difference testing is described on the Mplus website.
MLMV, WLSMV,
    and ULSMV difference testing is done using the DIFFTEST option.

RMSEA (Root Mean Square Error Of Approximation)
```

| | |
|---|---|
| Estimate | 0.029 |
| 90 Percent C.I. | 0.000 0.065 |
| Probability RMSEA <= .05 | 0.797 |

CFI/TLI

| | |
|---|---|
| CFI | 0.964 |
| TLI | 0.941 |

Chi-Square Test of Model Fit for the Baseline Model

| | |
|---|---|
| Value | 136.690 |
| Degrees of Freedom | 28 |
| P-Value | 0.0000 |

WRMR (Weighted Root Mean Square Residual)

| | |
|---|---|
| Value | 0.609 |

MODEL RESULTS

| | Estimate | S.E. | Est./S.E. | Two-Tailed P-Value |
|---|---|---|---|---|
| SES BY | | | | |
| Q2 | 1.000 | 0.000 | 999.000 | 999.000 |
| R | 0.412 | 0.093 | 4.445 | 0.000 |
| S | 0.652 | 0.214 | 3.044 | 0.002 |
| T | 0.119 | 0.026 | 4.502 | 0.000 |
| C10C ON | | | | |
| SES | 0.023 | 0.051 | 0.448 | 0.654 |
| E ON | | | | |
| SES | -0.037 | 0.057 | -0.650 | 0.516 |
| ZZ4 ON | | | | |
| SES | 0.027 | 0.037 | 0.745 | 0.456 |
| E ON | | | | |
| C10C | -0.068 | 0.101 | -0.672 | 0.501 |
| ZZ4 ON | | | | |
| B2_IMP | 0.039 | 0.011 | 3.472 | 0.001 |
| E | -0.090 | 0.069 | -1.313 | 0.189 |
| C10C | -0.137 | 0.060 | -2.293 | 0.022 |
| Intercepts | | | | |
| Q2 | 6.768 | 1.364 | 4.964 | 0.000 |
| R | 2.515 | 0.620 | 4.059 | 0.000 |
| T | 2.594 | 0.220 | 11.786 | 0.000 |
| ZZ4 | -0.351 | 0.210 | -1.670 | 0.095 |

| | | | | |
|---|---|---|---|---|
| Thresholds | | | | |
| E$1 | 0.371 | 0.667 | 0.557 | 0.578 |
| E$2 | 0.560 | 0.660 | 0.849 | 0.396 |
| E$3 | 0.766 | 0.665 | 1.152 | 0.249 |
| S$1 | -1.425 | 0.709 | -2.009 | 0.044 |
| S$2 | 1.767 | 0.707 | 2.501 | 0.012 |
| C10C$1 | 0.577 | 0.554 | 1.041 | 0.298 |
| C10C$2 | 2.607 | 0.560 | 4.652 | 0.000 |
| C10C$3 | 2.999 | 0.622 | 4.824 | 0.000 |
| Variances | | | | |
| SES | 3.129 | 1.025 | 3.052 | 0.002 |
| Residual Variances | | | | |
| Q2 | 5.157 | 0.749 | 6.887 | 0.000 |
| R | 1.239 | 0.154 | 8.038 | 0.000 |
| T | 0.234 | 0.018 | 13.084 | 0.000 |
| ZZ4 | 0.699 | 0.053 | 13.302 | 0.000 |

STANDARDIZED MODEL RESULTS

STDYX Standardization

| | Estimate | S.E. | Est./S.E. | Two-Tailed P-Value |
|---|---|---|---|---|
| SES BY | | | | |
| Q2 | 0.615 | 0.071 | 8.670 | 0.000 |
| R | 0.547 | 0.069 | 7.912 | 0.000 |
| S | 0.756 | 0.083 | 9.064 | 0.000 |
| T | 0.399 | 0.060 | 6.694 | 0.000 |
| C10C ON | | | | |
| SES | 0.040 | 0.089 | 0.453 | 0.651 |
| E ON | | | | |
| SES | -0.065 | 0.097 | -0.664 | 0.507 |
| ZZ4 ON | | | | |
| SES | 0.056 | 0.074 | 0.758 | 0.448 |
| E ON | | | | |
| C10C | -0.067 | 0.100 | -0.676 | 0.499 |
| ZZ4 ON | | | | |
| B2_IMP | 0.113 | 0.033 | 3.388 | 0.001 |
| E | -0.106 | 0.080 | -1.317 | 0.188 |
| C10C | -0.160 | 0.069 | -2.310 | 0.021 |
| Intercepts | | | | |
| Q2 | 2.351 | 0.481 | 4.892 | 0.000 |

| | | | | |
|---|---|---|---|---|
| R | 1.891 | 0.482 | 3.925 | 0.000 |
| T | 4.922 | 0.471 | 10.440 | 0.000 |
| ZZ4 | -0.408 | 0.239 | -1.712 | 0.087 |
| **Thresholds** | | | | |
| E$1 | 0.370 | 0.664 | 0.557 | 0.577 |
| E$2 | 0.557 | 0.657 | 0.849 | 0.396 |
| E$3 | 0.762 | 0.661 | 1.152 | 0.249 |
| S$1 | -0.934 | 0.441 | -2.116 | 0.034 |
| S$2 | 1.157 | 0.441 | 2.625 | 0.009 |
| C10C$1 | 0.577 | 0.554 | 1.041 | 0.298 |
| C10C$2 | 2.604 | 0.560 | 4.654 | 0.000 |
| C10C$3 | 2.997 | 0.621 | 4.826 | 0.000 |
| **Variances** | | | | |
| SES | 1.000 | 0.000 | 999.000 | 999.000 |
| **Residual Variances** | | | | |
| Q2 | 0.622 | 0.087 | 7.145 | 0.000 |
| R | 0.700 | 0.076 | 9.247 | 0.000 |
| T | 0.841 | 0.048 | 17.683 | 0.000 |
| ZZ4 | 0.950 | 0.028 | 33.461 | 0.000 |

R-SQUARE

| Observed Variable | Estimate | S.E. | Est./S.E. | Two-Tailed P-Value | Scale Factors |
|---|---|---|---|---|---|
| E | 0.009 | 0.020 | 0.456 | 0.648 | 0.995 |
| Q2 | 0.378 | 0.087 | 4.335 | 0.000 | |
| R | 0.300 | 0.076 | 3.956 | 0.000 | |
| S | 0.571 | 0.126 | 4.532 | 0.000 | 0.655 |
| T | 0.159 | 0.048 | 3.347 | 0.001 | |
| ZZ4 | 0.050 | 0.028 | 1.776 | 0.076 | |
| C10C | 0.002 | 0.007 | 0.226 | 0.821 | 0.999 |

QUALITY OF NUMERICAL RESULTS

Condition Number for the Information Matrix 0.175E-03
(ratio of smallest to largest eigenvalue)

TOTAL, TOTAL INDIRECT, SPECIFIC INDIRECT, AND DIRECT EFFECTS

| | Estimate | S.E. | Est./S.E. | Two-Tailed P-Value |
|---|---|---|---|---|
| **Effects from SES to ZZ4** | | | | |
| Total | 0.028 | 0.038 | 0.739 | 0.460 |
| Total indirect | 0.000 | 0.008 | 0.039 | 0.969 |

```
  Specific indirect

    ZZ4
    E
    SES                0.003      0.006      0.602      0.547

    ZZ4
    C10C
    SES               -0.003      0.007     -0.446      0.656

    ZZ4
    E
    C10C
    SES                0.000      0.000      0.353      0.724

  Direct
    ZZ4
    SES                0.027      0.037      0.745      0.456


Effects from C10C to ZZ4

  Total               -0.131      0.060     -2.170      0.030
  Total indirect       0.006      0.010      0.595      0.552

  Specific indirect

    ZZ4
    E
    C10C               0.006      0.010      0.595      0.552

  Direct
    ZZ4
    C10C              -0.137      0.060     -2.293      0.022


STANDARDIZED TOTAL, TOTAL INDIRECT, SPECIFIC INDIRECT, AND DIRECT EFFECTS


STDYX Standardization

                                                    Two-Tailed
                    Estimate       S.E.  Est./S.E.    P-Value

Effects from SES to ZZ4

  Total                0.057      0.076      0.752      0.452
  Total indirect       0.001      0.017      0.039      0.969

  Specific indirect

    ZZ4
    E
```

```
    SES                 0.007      0.011      0.611      0.541

    ZZ4
    C10C
    SES                -0.006      0.014     -0.449      0.654

    ZZ4
    E
    C10C
    SES                 0.000      0.001      0.355      0.723

  Direct
    ZZ4
    SES                 0.056      0.074      0.758      0.448


Effects from C10C to ZZ4

  Total                -0.153      0.070     -2.184      0.029
  Total indirect        0.007      0.012      0.596      0.551

  Specific indirect

    ZZ4
    E
    C10C                0.007      0.012      0.596      0.551

  Direct
    ZZ4
    C10C               -0.160      0.069     -2.310      0.021


RESIDUAL OUTPUT


     ESTIMATED MODEL AND RESIDUALS (OBSERVED - ESTIMATED)


           Model Estimated Means/Intercepts/Thresholds
              E$1           E$2           E$3           Q2            R
              ________      ________      ________      ________
________
      1         0.370         0.557         0.762         6.768
2.515


           Model Estimated Means/Intercepts/Thresholds
              S$1           S$2           T             ZZ4
C10C$1
              ________      ________      ________      ________
________
      1        -0.934         1.157         2.594        -0.351
0.577
```

```
           Model Estimated Means/Intercepts/Thresholds
              C10C$2        C10C$3
              ________      ________
      1         2.604         2.997


           Residuals for Means/Intercepts/Thresholds
              E$1           E$2           E$3           Q2            R
              ________      ________      ________      ________
________
      1        -0.001         0.000        -0.001         0.001
0.000


           Residuals for Means/Intercepts/Thresholds
              S$1           S$2           T             ZZ4
C10C$1
              ________      ________      ________      ________
________
      1        -0.001         0.003         0.000         0.000        -
0.002


           Residuals for Means/Intercepts/Thresholds
              C10C$2        C10C$3
              ________      ________
      1         0.003         0.003


           Model Estimated Slopes
              B2_IMP
              ________
 E              0.000
 Q2             0.000
 R              0.000
 S              0.000
 T              0.000
 ZZ4            0.039
 C10C           0.000


           Residuals for Slopes
              B2_IMP
              ________
 E             -0.012
 Q2            -0.126
 R             -0.002
 S              0.010
 T             -0.037
 ZZ4            0.000
 C10C           0.020
```

Model Estimated Covariances/Correlations/Residual Correlations

| | E | Q2 | R | S | T |
|---|---|---|---|---|---|
| E | | | | | |
| Q2 | -0.119 | 8.286 | | | |
| R | -0.049 | 1.288 | 1.769 | | |
| S | -0.051 | 1.336 | 0.550 | | |
| T | -0.014 | 0.372 | 0.153 | 0.159 | 0.278 |
| ZZ4 | -0.084 | 0.087 | 0.036 | 0.037 | 0.010 |
| C10C | -0.070 | 0.071 | 0.029 | 0.030 | 0.008 |

Model Estimated Covariances/Correlations/Residual Correlations

| | ZZ4 | C10C |
|---|---|---|
| ZZ4 | 0.727 | |
| C10C | -0.129 | |

Residuals for Covariances/Correlations/Residual Correlations

| | E | Q2 | R | S | T |
|---|---|---|---|---|---|
| E | | | | | |
| Q2 | -0.111 | 0.004 | | | |
| R | 0.014 | -0.092 | 0.000 | | |
| S | -0.018 | 0.072 | -0.010 | | |
| T | 0.026 | -0.004 | 0.021 | -0.015 | 0.000 |
| ZZ4 | 0.000 | -0.141 | 0.026 | 0.030 | 0.013 |
| C10C | 0.000 | -0.029 | -0.118 | 0.078 | 0.017 |

Residuals for Covariances/Correlations/Residual Correlations

| | ZZ4 | C10C |
|---|---|---|
| ZZ4 | 0.000 | |
| C10C | 0.000 | |

UNIVARIATE PROPORTIONS FOR CATEGORICAL VARIABLES

| | Observed | Estimated | Residual (Observed-Estimated) |
|---|---|---|---|
| E | | | |
| Category 1 | 0.713 | 0.644 | 0.069 |

```
       Category 2         0.060          0.067         -0.007
       Category 3         0.057          0.066         -0.009
       Category 4         0.170          0.223         -0.053
     S
       Category 1         0.136          0.175         -0.039
       Category 2         0.704          0.701          0.003
       Category 3         0.160          0.124          0.036
     C10C
       Category 1         0.600          0.718         -0.118
       Category 2         0.389          0.277          0.111
       Category 3         0.007          0.003          0.004
       Category 4         0.004          0.001          0.002
```

MODEL MODIFICATION INDICES

NOTE:  Modification indices for direct effects of observed dependent variables
regressed on covariates and residual covariances among observed dependent
variables may not be included.  To include these, request MODINDICES
(ALL).

Minimum M.I. value for printing the modification index    10.000

```
                                   M.I.     E.P.C.  Std E.P.C.  StdYX
E.P.C.

BY Statements

SES      BY Q2                   999.000     0.000      0.000
0.000

ON/BY Statements

SES      ON SES      /
SES      BY SES                  999.000     0.000      0.000
0.000

ON Statements

C10C     ON C10C                 999.000     0.000      0.000
0.000
```

DIAGRAM INFORMATION

  Use View Diagram under the Diagram menu in the Mplus Editor to view the diagram.
  If running Mplus from the Mplus Diagrammer, the diagram opens automatically.

  Diagram output

```
e:\@erika_thomaz\@a_ufma\a_orientacoes\elisa_miranda\mestrado\artigo_rant
es\original_plos.dgm

     Beginning Time:  14:06:48
        Ending Time:  14:06:48
       Elapsed Time:  00:00:00
```

MUTHEN & MUTHEN
3463 Stoner Ave.
Los Angeles, CA  90066

Tel: (310) 391-9971
Fax: (310) 391-8971
Web: www.StatModel.com
Support: Support@StatModel.com

Copyright (c) 1998-2014 Muthen & Muthen
